# 199 Concordance between Emerging Infections Program and Centers for Medicare and Medicaid Services data for patients in Nashville, TN, 2022

**DOI:** 10.1017/ash.2026.10587

**Published:** 2026-06-23

**Authors:** Mary “Ellen” Acree, Sandra Naegele, Lionel Sielatchom Noubissie, Jacob Barnhart

**Affiliations:** 1 Endeavor Health; 2 NorthShore University HealthSystem

## Abstract

**Background:** Pyogenic arthritis is a commonly encountered condition by Infectious Diseases clinicians and can occur in native joints as well as prosthetic joints. Synovial fluid culture is key in identifying the pathogen but there is a high incidence of culture-negative infections – up to 40% in native pyogenic arthritis and up to 15% in prosthetic joint infections. Multiplex polymerase chain reaction (PCR) testing offers the potential for rapid organism identification but real-world data on utilization of the test is lacking. **Methods:** A health system in the Chicagoland area recently began use of the BIOFIRE® Joint Infection Panel, which tests for 39 common bacterial and fungal causes of joint infection, as well as several markers of antimicrobial resistance. If the PCR is ordered, a synovial fluid culture must also be ordered. Of note, coagulase-negative staphylococci and Cutibacterium acnes are not targets on the PCR panel. A retrospective review was conducted of synovial fluid PCR testing among 5 hospitals from 1/1/25 – 12/6/25. **Result:** A total of 168 synovial fluid PCR tests were sent and 48 (28.4%) were positive. Age, gender, and race/ethnicity were similar between the PCR-positive and PCR-negative groups. In the PCR-positive and PCR-negative groups, 54.2% and 57.5% of patients had a prosthetic joint, respectively. All patients also had a synovial fluid culture obtained. Among patients with a positive PCR test, PCR and culture results were concordant for 31 (64.6%) patients. Of the 17 patients with a positive PCR but a discordant culture result, 14 had a negative synovial fluid culture and 3 had a culture identifying the organism found on PCR but also a second bacteria (Staphylococcus epidermidis, C. acnes, Finegoldia magna). Additionally, 90.8% of negative PCR tests had a corresponding negative culture result. Among the 12 patients with a negative PCR and a positive culture, six (50%) had growth of organisms included on the PCR panel (Staphylococcus aureus [n=2], Pseudomonas aeruginosa [n=2], Escherichia coli [n=1], Streptococcus infantarius [n=1]). The remaining isolates (Pasteurella multocida, Corynebacterium striatum, S. epidermidis [n=2], Dermabacter hominis, and Haemophilus parainfluenzae) were off-panel organisms. **Conclusion:** Synovial fluid PCR paired with culture can decrease the incidence of pyogenic arthritis without an identifiable pathogen among patients with native and prosthetic joint infections. Further research should examine the impact of the PCR test on antibiotic use, including time to targeted therapy, which may inform diagnostic stewardship recommendations regarding the test.

